# The agency domain and behavioral interactions: assessing positive animal welfare using the Five Domains Model

**DOI:** 10.3389/fvets.2023.1284869

**Published:** 2023-11-02

**Authors:** Katherine E. Littlewood, Morgan V. Heslop, Mia L. Cobb

**Affiliations:** ^1^Animal Welfare Science and Bioethics Centre, School of Veterinary Science, Massey University, Palmerston North, New Zealand; ^2^Animal Welfare Science Centre, Faculty of Science, The University of Melbourne, Melbourne, VIC, Australia

**Keywords:** agency, animal welfare, positive animal welfare, positive affective engagement, quality of life, good life, happiness, animal wellbeing

## Abstract

Animal welfare denotes how an animal experiences their life. It represents the overall mental experiences of an animal and is a subjective concept that cannot be directly measured. Instead, welfare indicators are used to cautiously infer mental experiences from resource provisions, management factors, and animal-based measures. The Five Domains Model is a holistic and structured framework for collating these indicators and assessing animal welfare. Contemporary approaches to animal welfare management consider how animals can be given opportunities to have positive experiences. However, the uncertainty surrounding positive mental experiences that can be inferred has resulted in risk-averse animal welfare scientists returning to the relative safety of positivism. This has meant that aspects of positive welfare are often referred to as animal ‘wants’. Agency is a concept that straddles the positivist-affective divide and represents a way forward for discussions about positive welfare. Agency is the capacity of individual animals to engage in voluntary, self-generated, and goal-directed behavior that they are motivated to perform. Discrete positive emotions are cautiously inferred from these agentic experiences based on available knowledge about the animal’s motivation for engaging in the behavior. Competence-building agency can be used to evaluate the potential for positive welfare and is represented by the Behavioral Interactions domain of the Five Domains Model. In 2020, The Model was updated to, amongst other things, include consideration of human-animal interactions. The most important aspect of this update was the renaming of Domain 4 from “Behavior” to “Behavioral Interactions” and the additional detail added to allow this domain’s purpose to be clearly understood to represent an animal’s opportunities to exercise agency. We illustrate how the Behavioral Interactions domain of The Model can be used to assess animals’ competence-building agency and positive welfare. In this article, we use the examples of sugar gliders housed in captivity and greyhounds that race to illustrate how the agentic qualities of choice, control, and challenge can be used to assess opportunities for animals to exercise agency and experience positive affective engagement.

## 1. Introduction

Animal welfare is both an academic discipline and a property of sentient animals. Animal welfare has been described as multi-disciplinary (1); however, it is increasingly becoming a trans-disciplinary field as it draws from and interacts across disciplines such as animal welfare science (including neurophysiology, applied ethology, and animal science), animal ethics (including philosophy and bioethics), psychology (including beliefs and attitudes, social psychology, and human behavior change), education, communication, animal law, and policy.

As a property of sentient animals, animal welfare represents how an animal experiences their life. Animal welfare, in this context, is a state within an animal. There are myriad definitions used to express this sentiment. However, the most consistently important concept for an animal is a focus on its subjective mental experiences. These mental experiences can vary from positive (e.g., pleasure from a comfortable environment, companionship from conspecifics, feeling well-fed) to negative (e.g., discomfort due to thermal extremes, loneliness, and a feeling of thirst) and can change over time (2). Added to this understanding that mental experiences matter to an animal, those mental experiences hold ethical relevance to the people who interact with animals (3, 4). Mental experiences underpin many animal laws [e.g., (5, 6)] that focus on preventing unnecessary or unreasonable suffering (i.e., suffering is a catch-all term for a range of negative mental experiences). A methodology for assessing animal welfare that focuses on an animal’s mental experience is increasingly considered best practice in contemporary animal welfare science (1). This way of assessing animal welfare also creates unity within the discipline by aligning with the experiential focus of other facets (i.e., ethics, policy, and laws). In this article, an animal’s *welfare* refers to its overall mental (affective) experiences.

This way of understanding animal welfare can pose challenges when it comes to welfare assessment. Most importantly, mental experiences are felt by the individual animal – they are subjective – and cannot be directly measured. This can be difficult for those accustomed to measuring other quantifiable features of animals, such as reproductive success, body weight, or heart-rate variability. Scientists can find that stepping over Dawkins’ ‘bridge’ from the measurable and observable to the inferential and deducible makes them confront long-held beliefs and values (e.g., positivism) inherent in science [e.g., (2–4)]. However, affective neuroscience and studies in applied ethology allow us to make cautious inferences about relationships between measurable features of animals and their subjective mental experiences (7–13).

Animal welfare, conceptualized as the mental experiences of animals, can also make inferences about positive welfare challenging (14). Given that “good” animal welfare represents an overall positive welfare state, or a good life, for an animal (i.e., when opportunities for animals to have predominantly positive mental experiences are provided), how can positive welfare be assessed in a scientifically robust manner? We propose that the way forward is to consider animal agency.

Agency represents the new frontier in animal welfare assurance. While traditional animal welfare management has focused almost exclusively on minimizing animal welfare compromise, or “suffering,” contemporary approaches consider how animals can be given opportunities to experience positive welfare (3, 14–17). For example, standards of care have historically focused on security and physical health aspects of animal housing environments. Guidelines for dairy cattle specify, “Cattle without shelter need to put more energy into normal functioning and less into production” (18). Whereas modern standards now include additional consideration for the positive mental experience of animals, with provisions relating to bedding, cleaning, lighting, temperature, noise, ventilation, and humidity [e.g., (19)]. This is to ensure that animals do not only avoid discomforts that may be harmful but will be comfortable. More recently, positive animal welfare has been characterized by four features: positive emotions; positive affective engagement; quality of life; and happiness (14). We argue that each of these features can be linked to animal agency. More specifically, these features are more likely to occur when animals engage with opportunities to exercise agency.

Agency is the capacity of animals to engage in voluntary, self-generated, and goal-directed behavior that they are motivated to perform (20, 21). These behaviors can be motivated by positive affective consequences (22, 23). The collective term for these positive subjective mental experiences (or affects) resulting from reward-based motivations is “positive affective engagement” (23–25). This term reflects the engagement, or “flow,” inherent in these experiences (26). Animals are pleasantly occupied [e.g., a detection dog engaged in a scenting task (26, 27)] to such an extent that they can become oblivious to other sensations or mental experiences – provided they are not significantly negative (23–26). Discrete positive emotions, or affective states, are cautiously inferred from these agentic experiences based on available knowledge about the animal’s motivation for engaging in the behavior. Such motivations can be encoded at the species level and passed to the individual animal via their genome (phylogenetic) or occur at the individual animal level because of environmental interactions within the individual’s lifetime (ontogenetic). The exact nature of these drivers and their impact on affective experiences are, as yet, poorly understood.

For this reason, positive welfare, or more precisely, the uncertainty surrounding mental (affective) experiences that can be inferred, has resulted in risk-averse animal welfare scientists returning to the relative safety of positivism. This has meant that aspects of positive welfare are often referred to as animal “wants” – and “needs” are the basic provisions that precede these “wants” (28–32). Framing animal welfare as “needs” and “wants” risks reducing human responsibility towards animals to solely neutralizing negative experiences (“needs”), while positive experiences (“wants”) could be perceived as an optional luxury (33, 34). Agency is a concept that straddles the positivist-affective divide and represents a way forward for productive discussions about positive animal welfare and to help advance the welfare of animals under human care.

This article aims to articulate how agency can be used to assess animal welfare and the relationship between an animal’s welfare and their ability to exercise agency. A secondary objective is to illustrate how the Behavioral Interactions domain (Domain 4) of the Five Domains Model represents this expression of agency.

## 2. The Five Domains Model and animal welfare assessment

When understood in affective state terms (i.e., a focus on mental experiences), animal welfare should be assessed in such terms (1). The Five Domains Model is a framework for assessing animal welfare that focuses on subjective mental experiences that matter to the animal (35). Other animal welfare assessment frameworks exist. For example, Welfare Quality focuses on four areas: good feeding, good housing, good health, and appropriate behavior (36). However, none focus on the mental experiences of animals to the same extent as the Five Domains Model (35).

**Figure 1:**
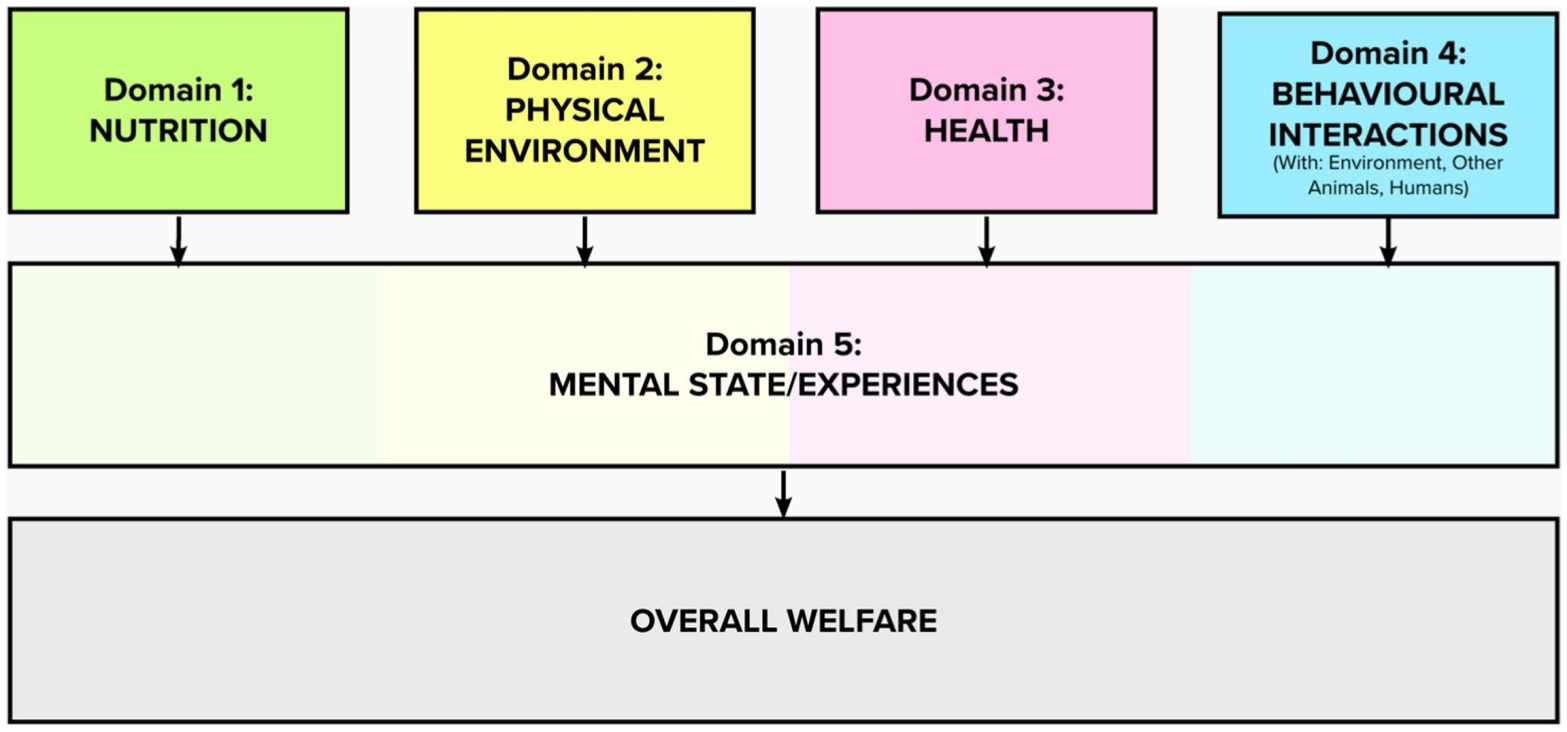
The 2020 Five Domains Model of animal welfare.

The structure of the Five Domains Model is illustrated in Figure 1. The first four domains represent inputs to the animal that are processed by their species-specific physiology and behavioral biology resulting in physical/functional states (Domains 1 to 3) or representing an animal’s externally perceived situation (Domain 4) (35).

Domain 1 (Nutrition) and Domain 3 (Health) are the physical/functional states of the animal (e.g., nutritional or hydration status and physical health issues such as illness and physical dysfunction) that are the states most familiar to veterinary and animal scientists (37). Domain 2 (Physical Environment) focuses on conditions available to the animal (e.g., space allowance, air quality, bedding). Domain 4 (Behavioral Interactions) represents the animal’s ability to exercise agency in their interactions with the environment, other animals, and humans (35).

The Model is a framework and focusing device for animal welfare assessment that needs to be operationalized for the specific context and animal The Model is used to assess (38). Valid welfare indicators need to be established for each of the states/conditions/agency initiatives in Domains 1 to 4. The second part of this two-step process requires that these welfare indicators be validated for the specific mental experience they can infer in Domain 5, Mental State (39).

Domain 5 (Mental State) represents the animal’s overall welfare, or lived experience, in affective terms (35). This domain is not assessed separately, but rather it reminds users to draw affective inferences from states/conditions/agency initiatives identified in Domains 1 to 4. In this way, The Model takes an affective state approach to animal welfare assessment (35). Welfare impacts identified in Domains 1 to 4 must have corresponding mental experiences (inferred in Domain 5) that matter to the animal to impact their welfare (35).

Evidence from multiple disciplines (e.g., affective neuroscience, physiology, ethology, psychology) informs The Model’s use and subsequent updates. In 2020, The Model was updated to, amongst other things, include consideration of human-animal interactions (35). The most important aspect of this update was the renaming of Domain 4 from “Behavior” to “Behavioral Interactions” and the additional detail added to this domain to allow its purpose to be more clearly understood. This domain had been understood by its authors as “The Agency Domain” for several years preceding this update. However, 2020 marked the year where there was a recognized need for Domain 4 to be renamed to link it more explicitly to an animal’s ability to exercise agency (35). It was envisaged that this update would help readers better understand Domain 4 and the important role of animal agency in animal welfare assessment (15).

## 3. Behavioral interactions and domain alignment

Renaming Domain 4 of The Model to “Behavioral Interactions” (35) in 2020 was necessary to align it with the “input” focus of Domains 1 to 3. Domain 1, Nutrition, focuses on nutritional inputs (e.g., food and water provision) that may impact the animal’s nutritional status in functional terms. Domain 2, Physical Environment, inputs are externally available conditions in the physical environment (e.g., ambient temperature, air quality). Domain 3, Health, is used for factors contributing to vitality, disease, injury, or other functional or physiological conditions contributing to an animal’s physical health and fitness (e.g., parasite control, vaccination). Overall, Domains 1 to 3 focus users on various survival-related inputs and provide a structured approach to inferring how these inputs, and their effects on physical/functional states or available conditions, impact overall welfare (mental experiences) in Domain 5, Mental State (35).

Before the 2020 update, Domain 4 was called “Behavior” and was routinely used to describe an animal’s outward behavioral expression. However, behavior is an indicator of welfare. Behaviors can be used across all four domains (e.g., shade-seeking behavior may be used in Domain 2 to evaluate the suitability of the Physical Environment an animal is kept within). The updated term ‘Behavioral Interactions’ focuses on inputs to the animal that constrain or provide opportunities for animals to exercise agency (40). Three subcategories were included to encourage users to consider opportunities for animals to exercise agency during interactions with: (A) the environment; (B) other animals; and (C) humans (35).

Domain 2 was also renamed in 2020 from “Environment” to “Physical Environment” to clarify and help distinguish it from Domain 4 (35). Domain 2 focuses on provisions and aspects of the environment that contribute to an animal’s physical comfort. In contrast, Domain 4 (specifically the subcategory of ‘interactions with the environment’) focuses on parts of the environment an animal interacts with and the ways an animal interacts with these features (35).

### 3.1. Environmental enrichment

Behavioral Interactions (Domain 4) is where environmental enrichment is considered (35, 37) within the Five Domains Model. Environmental enrichment refers to structures and stimuli that promote species-specific behavior that is important and beneficial from the perspective of an individual (41). This means that environmental enrichment broadly corresponds to features that give animals opportunities to exercise agency. Different types of enrichment have been articulated: occupational, physical, sensory, nutritional, and social (42). However, environmental enrichment can be difficult to apply when aligned with the Five Domains Model and the affective state orientation to animal welfare. Firstly, enrichment types are not all ‘environmental’ in their application. Occupational enrichment can result from interactions animals have with other animals, humans, or even smart technologies (43). Social enrichment, by definition, occurs during interactions with other animals and humans. When using Domain 4, it may be more beneficial to align enrichment types with the different sub-categories of this domain: Environment, Other Animals, and Humans (Table 1).

Operationalizing the term “enrichment” can be challenging. Environmental enrichment originated in laboratory animal welfare as a compensatory device but has become an increasingly essential tool for providing animals in managed captive settings with opportunities for positive welfare (44–46). Environmental enrichment is now used across zoos and aquaria (44, 47) and is increasingly reported in other settings (e.g., farm animals with enrichment opportunities such as brushes and showers). Environmental enrichment has undoubtedly led to improved animal welfare (48). However, it may have reached a point where the term ‘enrichment’ no longer aligns with contemporary animal welfare science thinking.

Enrichment implies an optional improvement that can be used in any setting to improve animal welfare. However, animals experiencing significantly negative mental experiences, for example, those raised in isolated and barren environments that do not provide agentic opportunity for social and exploratory behaviors, may be unable to respond to environmental enrichment features [e.g., captive bottlenose dolphins isolated in quarantine did not engage with enrichment toys (49)] (15). Enrichment cannot be treated as a panacea for all issues of welfare compromise or to legitimize housing animals in unsuitable conditions. Instead, there is a need to assess an animal’s welfare systematically and holistically across multiple domains to understand the best way(s) to optimize their welfare. For this reason, a more appropriate way forward may be to rephrase this concept as ‘environmental optimisation’ or ‘environmental challenge’ (21). Optimisation is more nuanced and implies a greater understanding of the underlying animal welfare compromise and the targeted strategies that should be developed to ameliorate it and bring about welfare improvement.

As a term, *environmental enrichment* has become synonymous with welfare improvement and is entrenched in many people’s minds. Thus, reframing its meaning may be a more effective way forward rather than changing the term. Fernandez argues that environmental enrichment was never meant solely to provide animals with *objects. Instead, it* refers to stimuli and/or events that result in animals having opportunities for enriched *quality of interactions* with their environment, other animals, and humans (50). Positive reinforcement training can modify these interactions and function as an enrichment [e.g., training promoted social interactions by moderating chimpanzee aggression during feeding (51)] (50). This framing aligns with the concept of agency and the interaction subcategories of Domain 4. The structured framework of The Model can be used to identify specific enriching interactions and then direct carefully considered and targeted interventions (35).

## 4. The agency domain and animal welfare

Agency is the capacity of animals to engage in voluntary, self-generated, and goal-directed behavior that they are motivated to perform (20, 21). These behaviors can be motivated by positive affective consequences, i.e., those that result in positive affective engagement, or by negative affective consequences (e.g., avoiding predation or other situations perceived as a threat) (7, 8, 52). Špinka describes three ways to understand the welfare benefits of animals having the capacity for agency: adaptive functioning, affective functioning, and awareness/selfhood (52). From the adaptive point of view, goal-directed behavior confers a survival advantage to animals. An animal that approaches interactions (with its environment, other animals, and/or humans) reactively or reflexively [e.g., the starfish has a righting reflex in response to inversion (53)] is less likely to survive in complex environments than one that has the cognitive capacity to be proactive (flexible) in its interactions (52). For example, wild deer fawns with mothers who proactively hid their young were more likely to survive in open habitats than reactive-mothered fawns (54). Conversely, expressing agency may be less critical to animals in simple environments with relatively stable interactions.

The affective functioning viewpoint focuses on evidence from affective neuroscience and an appreciation of the neurobiological mechanisms underpinning mental (affective) experiences (52). Fundamental to this viewpoint is the understanding that mental experiences are motivational forces (or drivers) for the complex behaviors animals perform (16). In other words, mental experiences are proximate causes of complex, but not reflexive, behavior (55, 56). More complex agentic capacities require more diverse underlying mental experiences. Animals operating competently within complex environments might be expected to possess a greater range of mental qualities because of a need to exercise greater agency.

Differing levels of awareness is another way of conceptualizing the welfare benefits of animal agency. In this conceptualisation, consciousness or self-awareness accumulates at different levels (52). The most basic level of awareness includes a sense of ‘core self’ that allows individuals to identify sensations and behaviors as their own in the present moment (7). The next awareness level relates to competence-building (57). At this level, the animal has the capacity for cognitive processes such as learning and memory, enabling them to accumulate skills and knowledge from previous experiences. In other words, animals can build competence towards a species-specific level of awareness when given opportunities to exercise agency. Long-term goals and aspirations are features of the highest awareness level and result from decision-making based on introspection (52). An animal’s umwelt, or unique perceptual world, is dictated by its awareness level (58). Therefore, a higher level of awareness gives a broader scope for umwelt.

**Figure 2:**
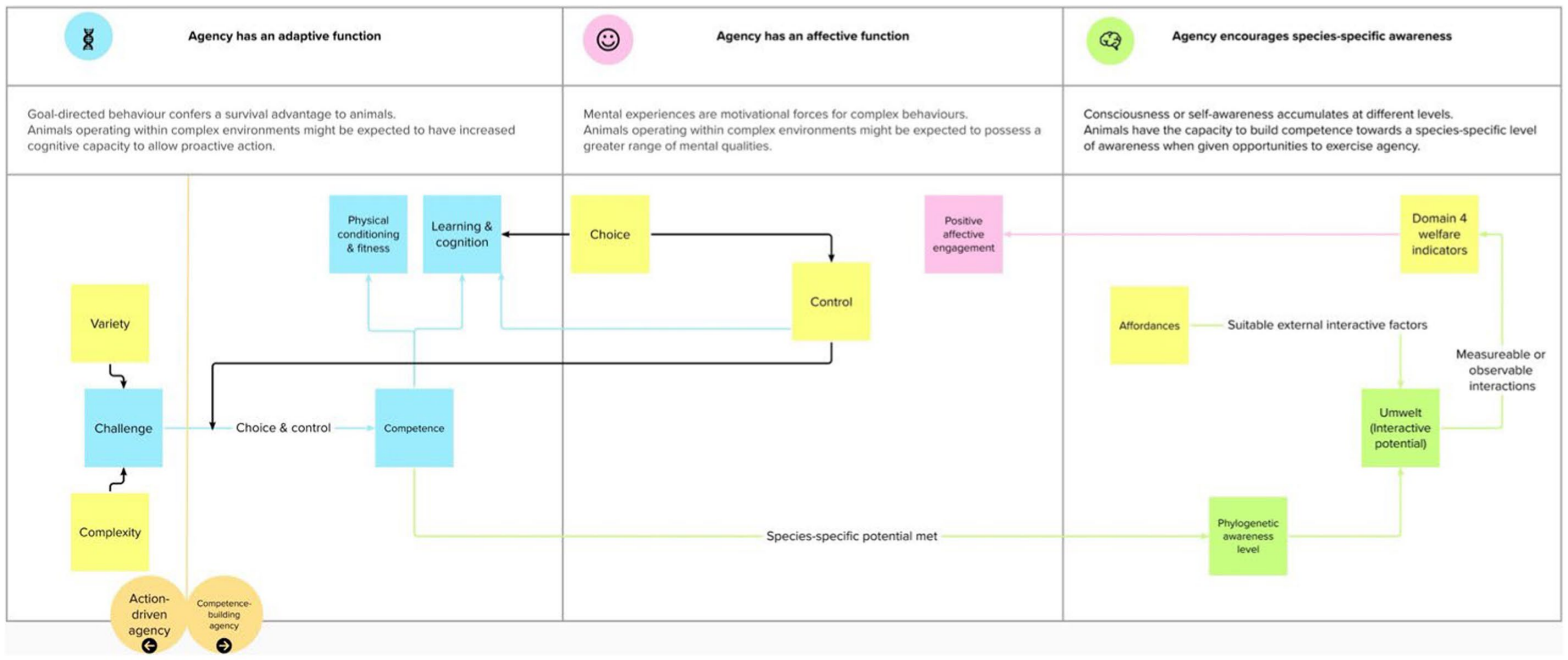
The three ways Špinka (52) relates agency to positive welfare and their relationship to other concepts used in animal welfare science.

Overall, Špinka identifies three ways agency relates to positive animal welfare (52). First, agency can be competence-building, and animals given opportunities to exercise agency are more likely to develop the skills (e.g., physical strength, social cohesion, mental resilience) necessary to overcome future agentic challenges. In other words, animals learn when they can exercise agency. Play in young animals is an example of this agentic learning process (59). Second, animals with opportunities to exercise agency can also experience positive affective engagement (i.e., a range of positive mental experiences), for example, pleasure, affectionate sociability, and care (15, 38). Finally, it is proposed that competence-building is welfare-enhancing as it supports the development of species-specific higher levels of awareness and allows an animal’s full interactive potential, and umwelt, to be met (52). At a higher level, this could result in animals, with the phylogenetic capacity, attributing meaning to their lives – a feature used to classify human happiness (52, 60), refer Figure 2.

### 4.1. Competence

A detailed exploration of agentic qualities such as competency, choice, control, challenge, and umwelt can further articulate agency. Competence results when an animal has the tools and strategies to deal with novel and ongoing challenges (31, 52, 61, 62). In other words, competency is the outcome of animals’ opportunities to exercise agency during their lifetime. The strategies for behavioral interactions (with the environment, other animals, and humans) have developed because of these opportunities, i.e., competence is agency-driven ontogenetic development (31, 57). Competence can enable future agency and be an outcome of exercising agency. The characteristics and skills developed during opportunities to exercise agency can enable animals to act with self-determination and increase their opportunities for agency (57).

Enhanced functional (e.g., physical conditioning) and cognitive (i.e., learned) capacities contribute to competence. Lack of space or incentive to exercise vigorously can result in poor physical conditioning, often exacerbated by uniform and limited opportunities for interactions with the environment (63). An individual animal unable to satisfy its genetic (phylogenetic) and developmental (ontogenetic) competence potential may experience a form of learned helplessness (63). Conversely, an animal in a barren environment may have developed less competence and a reduced threshold to perceive novelty (64). This could lead to arguments against providing animals in captivity with environmental enrichment opportunities. However, individuals with low competence living in captive environments should still be provided opportunities to exercise agency.

Suppose environmental enrichment provides opportunities for animals to exercise agency and develop competency. For example, they may become more challenging animals to contain in a zoo setting. In that case, a potential solution is to restrict opportunities for agency (e.g., by withholding enrichment) to limit escalating enrichment requirements. However, agency is required for animals to develop optimal physical functioning (e.g., via play) as well as mental capacities (26, 34, 65). Agency is also self-fulfilling and provides animals with opportunities to experience positive affective engagement in novel ways or ways that cannot otherwise be provided (15, 38). And even with restricted opportunities to exercise agency, and thus blunted competence, many animals retain the pre-programmed genetic potential (i.e., motivation) for agency due to phylogenetic developmental events within their species (31, 65). Impeding agency is in and of itself a welfare compromise, independent of how lowered competency may influence the perception of further welfare-compromising conditions. Ethically, if we are aware of these agentic requirements of animals (i.e., a valid evidence base exists), people are morally obligated to provide them. We anticipate this obligation will feature increasingly in the safeguarding, welfare assurance, standards of care, regulations, and animal management legislation in the coming years.

### 4.2. Choice

Choosing between two or more options allows animals to exercise agency (52, 57, 61, 63). Agentic “freedom of choice” roughly aligns with one of the Five Freedoms; “freedom to express normal behavior” (66, 67). However, providing for choice requires animals to have uninhibited options that align with their species-specific motivations (68). This requires detailed knowledge of what is normal for a species to do (i.e., knowledge of their behavioral biology). The domestication process has changed the behavioral biology of some animals to such an extent that ‘normal’ may cease to exist at the species level (3). Comparisons to wild populations cannot always be relied upon as many domesticated species no longer resemble their wild ancestors (3). Also, there is still much to learn about the behavioral biology of a range of taxa (69, 70). Added to this, there can be marked differences in the preferences of individual animals (52, 63).

Consequently, even at the species level, “normal” behavior represents a generalization that may not be informative when assessing the welfare of an individual animal. Overall, these considerations make it difficult to predict the behaviors an animal may want the “freedom to” perform. Affording animals agentic choice offers more versatile options for positive welfare, such as using technologies (40), than providing animals with contexts to perform specific “normal” behaviors – when they are known. Additionally, animals may prefer fewer choices than those offered to them or may prefer to interact with a choice not offered in managed settings.

Active environmental enrichment represents an example of agentic choice. Active enrichment is something an animal engages with directly through agentic choice being provided (e.g., food hidden in a tree to be detected and secured). In contrast, passive enrichment is provided to the animal without agentic choice (e.g., music is played) and may be not be perceived as rewarding by the animal (65, 71).

### 4.3. Control

Choice and control are interrelated aspects of exercising agency. Control is realized when an animal can *consistently* and *predictably* make choices and obtain the outcomes they are motivated to achieve (61, 65, 72). When animals can actively decide when and how to interact with the environment, other animals, and humans, they have an element of control over their choices (52, 57, 65, 72). Inaction is as essential as action; an animal choosing not to interact (e.g., with a toy offered to it) exerts control over its actions and therefore exercises agency (57, 65). Perception of control, whether exercised or not, influences cognition and behavior in animals responding to challenging situations (62, 65).

Perceived control forms the basis of cooperative care protocols and animal consent (73). Cooperative care involves training animals to make informed choices (i.e., consent) about their management (74). These training protocols should allow animals to consent and withdraw their consent at any time. Chin rest is an example of a common consent behavior used in dogs (74). Informed choice involves some level of predictability (i.e., control) and allows animals to exercise agency by controlling what happens to them (74). For example, automated technology can enable dairy cows to control their engagement with mechanical grooming brushes (75). When an animal can exert control, they may be more likely to engage in challenging interactions and develop competence (65).

### 4.4. Challenge

Various complex interactions can challenge animals and encourage the development of problem-solving abilities that confer competence (21, 46, 64, 65). Novelty increases the variety of interactions an animal may have. An animal can be provided with difficulty by making situations or tasks challenging to analyze, understand, or solve such that learning occurs (57, 61). Physical challenges can also offer advantages to animals by improving physical conditioning and fitness (64). Care must be taken to ensure challenges are not too far beyond the competency level of the individual animal as this can have negative affective consequences, e.g., result in frustration or anxiety (64). Suppose these challenges do not far exceed an animal’s current competency level (i.e., they are surmountable). In that case, they offer an opportunity for the animal to exercise agency and experience positive affective engagement (15, 38). Examples of so-called ‘environmental enrichment’ challenges, and their alignment within Behavioral Interactions, Domain 4, are presented in Table 1.

**Table 1:** Types of environmental enrichment (42) and their alignment with sub-categories of Domain 4.

Enrichment aligned as behavioral interactions with…		
The Environment	Other animals	Humans
Occupational, e.g., cognitive (puzzles, activities), exercise (mechanical, run) Physical, e.g., enclosure (size, complexity), accessories (items) Sensory, e.g., visual (windows), auditory (vocalizations), olfactory Nutritional, e.g., delivery (frequency, schedule), type (novel, variety)	Occupational, e.g., cognitive (group activities), exercise Social, e.g., contact (conspecific/non-conspecific), non-contact (visual, auditory, olfactory)	Occupational, e.g., psychological (training activities), exercise Social, e.g., contact, non-contact (visual, auditory, olfactory)

### 4.5. Umwelt and affordances

How an individual animal feels about its competence also matters for its welfare. An animal’s umwelt represents its unique perceptual and effector world, i.e., an animal’s inner world (58). A higher level of awareness gives a broader scope for umwelt (52). The concept of umwelt has the added advantage of considering the differences in sensory worlds between animal taxa (58). Umwelt goes beyond sense organ physiology and considers how an animal responds to their situation and how these responses modify their perceptions of self and subsequent interactions with the environment, other animals, and humans (58).

In their discussion of animal communication, Parton and Marler (58) liken umwelt to Gibson’s theory of affordances, which describes the relationships between animals and their environments (76). Affordances of the environment are what it offers the animal, good or bad (76). An affordance is measured relative to the animal and is unique to that animal rather than measured in abstract physical properties (e.g., load-bearing force). Because affordance is interpreted relative to the perceiver (76, 77), an animal’s unique perceptual world (umwelt) will impact its perceived affordances (58). Gibson (76) suggests that an animal’s ecological niche is a set of affordances. A niche is how an animal lives and their role, rather than the habitat or where they live (76). An animal’s perceived affordances may determine their ability to develop competence and, in turn, impact how they can exercise agency.

Each of the terms interact to provide an overview of how animals might be provided opportunities to exercise agency to engage in voluntary, self-generated, and goal-directed behaviors that they are motivated to perform (20, 21), as depicted in Figure 2.

## 5. The agency domain in action: assessing animal welfare

Špinka’s four tiers of agency can help further articulate the role of animal competency when using the Five Domains Model to assess animal welfare. These tiers are passive/reactive agency, action-driven agency, competence-building agency, and aspirational agency (52). The tiers are distinguished by the type of behavioral interaction an animal has, which relates to the dominant brain structure(s) and awareness level(s) operating.

*Passive/reactive agency* is characterized by passive or reflexive reactionary behaviors resulting from external stimuli. Most are driven by homeostatic and sensory affective states involving the brainstem or corresponding neural substrate in non-mammalian animals (52). The resultant drives are probably subconscious and unlikely to play a role in animal welfare and assessment. For example, moon jellyfish (*Aurelia* sp.) dive in response to turbulence (78).

*Action-driven agency* involves emotional action systems at the subcortical level (52). The resultant behaviors are mostly survival-related, aimed at procuring food, seeking shelter, and avoiding predation. This tier aligns most with Domains 1 to 3 (Nutrition, Physical Environment, and Health) and is not the ‘interactive’ agency considered in Domain 4 (Behavioral Interactions).

*Competence-building agency* involves active behavioral interactions to build skills and acquire information for later use. This tier involves learning-related emotions at the level of the basal ganglia or corresponding neural substrate in non-mammalian animals (52). Such activities are future-focused and, rather than achieving immediate outcomes, allow animals to enhance skills and gather information (i.e., develop competence) for future use. Examples include instrumental and social learning, exemplified by contrafreeloading whereby animals choose to work for food over obtaining freely available food (50, 78). Inspective and inquisitive exploration, communication, and some forms of play also fall within this tier (52). This tier most closely aligns with the operational intent of Domain 4 (Behavioral Interactions). In other words, competence-building agency is the construct being assessed when the Behavioral Interactions with the environment, other animals, and people in Domain 4 is used as part of a holistic welfare assessment protocol.

*Aspirational agency* is driven by an animal’s neocortex and allows for complex interactive behaviors resulting from planning and goal setting. These often involve *affectively* guided planning and intentions to act (52). However, the evidence thus far suggests that this agency level is less prominent in non-human animals. Therefore, this level of agency is not currently considered within the Five Domains Model of animal welfare assessment but does encourage debate about how an animal’s time perception and planning may be considered in future updates to The Model.

Given the traditional focus of animal welfare science on the biological functioning orientation and alleviating welfare compromise (3, 79), we have amassed substantial information that contributes towards our understanding of negative mental experiences aligned with Nutrition, Physical Environment, and Health, Domains 1 to 3. Behavioral Interactions, represented in Domain 4, and their aligned mental experiences have proven more challenging to study empirically. This most likely stems from the difficulty scientists face when attempting to develop paradigms to evaluate agency robustly. This is particularly true for mental experiences traditionally assigned a positive valence (25, 34). However, as mentioned, we should avoid returning to the relative safety of positivism, where any reference to mental experiences is side-stepped. Instead, these challenges encourage us to exercise extra caution when considering mental experiences aligned with Behavioral Interactions and the expression of agency (Domain 4). Moving forward, animal welfare assessment using Domain 4 could be performed by reflecting on an animal’s ability to exercise various qualities of agency (see Section 4 of this paper) and aligning these to the experience of positive affective engagement (a catch-all term for positive mental experiences related to exercising competence-building agency) (15, 38). The terms ‘pleasure’ or ‘happiness’ could be used to reflect this when communicating with a lay audience.

### 5.1. Impediments to agency being exercised

Negative mental experiences inferred from impacts in Behavioral Interactions (Domain 4) result from impediments to an animal’s ability to exercise competence-building agency. These negative experiences reflect the cognitive responses of animals to being kept in impoverished environments (e.g., a laboratory rat in experimental deprivation conditions), under firm behavioral restriction (e.g., a working guide dog that cannot actively explore by sniffing or interact with other people or animals it encounters), or confronted by threatening situations (e.g., a horse kept with resource guarding conspecifics). This helps explain why these negative experiences have been collectively termed ‘situation-related negative affects’; they reflect the animal’s perception of their external circumstances, i.e., their situation (15, 35).

Impoverishment is a feature of restricted opportunities to engage in interactive behaviors – with the environment, other animals, or humans. Examples of these restrictions include limited space, barren or invariant features in enclosures, and social animals with little or no access to the company of others (15, 80). The development of negative mental experiences in restricted circumstances is believed to result from thwarted genetically pre-programmed (phylogenetic) or learned (ontogenetic) motivations to engage in rewarding behaviors or behaviors that result in a reward (7, 8, 15, 21). Such adverse experiences inferred (in Mental Experiences, Domain 5) from restricted circumstances may initially include frustration and fear (e.g., short-term kennelling of dogs) and then give way to boredom, depression, helplessness, loneliness, and isolation (8, 20). These latter mental experiences may promote low activity and energy conservation where resources are limited (81, 82). In other words, these mental experiences may result from loss or lack of reward following unsuccessful attempts to engage in highly motivated behaviors, i.e., when competence-building agency has been impeded.

Interactions (with the environment, other animals, and humans) that are cognitively perceived as threatening are also aligned with Domain 4, consistent with the positive and negative inputs possible in Domains 1–3 (Nutrition, Physical Environment, Health). Examples of potentially threatening situations include possible or actual attack, separation from the security and protection of others of social significance, and overstimulation or being presented with challenges that an animal has not developed competence to manage or avoid (15). Negative experiences inferred (in Domain 5, Mental State) from threatening situations may include anxiety, fear, and panic (8, 15). These negative mental experiences align with Mendl et al.’s upper left quadrant, i.e., Q4 of the functional core affect model, resulting from a desire to avoid aversive situations (81, 82). They promote coordinated responses to the presence of threat or danger. Such experiences are unlikely to be competence-building if the circumstances impede an animal’s ability to exercise agency through choice and control (e.g., victimization in a confined space).

### 5.2. Opportunities to exercise agency

Positive mental experiences inferred from Behavioral Interaction factors in Domain 4 are attributed to animals having opportunities to exercise agency and express more of their behavioral repertoire (15, 35). Correction of impacts in Nutrition, Physical Environment, and Health (Domains 1 to 3) that generate survival-related negative Mental Experiences (Domain 5) may enable the animal to refocus on engaging in rewarding behaviors. In other words, survival-related negative mental experiences at high intensities (i.e., compromised welfare) dominate the overall mental experiences of an animal, but when minimized, allow the animal to exercise agency and experience positive affective engagement (15, 38). This could be akin to an animal experiencing an overall feeling of physical safety when survival-related experiences aligned with Domains 1 to 3 are mitigated (83). Once physically safe, animals are more likely to engage in the rewarding Behavioral Interaction activities of Domain 4 (83).

Short-lived positive experiences may be generated from survival-related behaviors motivated by negative mental experiences (15). Water drinking behavior (Domain 1) initiated by the negative experience of thirst (Domain 5) may also result in transient positive experiences such as oral wetting and quenching pleasure (13). Such positive mental experiences may reduce or replace negative experiences but are unlikely to contribute to an overall positive welfare state long-term (15).

In contrast, some situation-related negative experiences may be replaced by positive ones when improvements are made to interactions (with the environment, other animals, and/or humans) that allow animals to engage in more rewarding behaviors (13, 17). For domestic species kept in human-dependent conditions, the negative experiences generated by such impeded interactions (i.e., impeded agency) often require intentional human intervention to correct. Again, providing opportunities to engage in rewarding behaviors is the basis of environmental enrichment strategies (44). Enrichment initiatives can serve to promote positive mental experiences (15, 38).

As mentioned in section 4.1, negative experiences (e.g., helplessness and isolation) can result from restricted circumstances (81, 82). Interventions to replace these negative experiences with positives (e.g., happy, excited) should focus on providing animals with opportunities to acquire rewarding experiences during their behavioral interactions (with the environment, other animals, and humans) (81, 82). Stimulus-rich and diverse or novel settings allow animals to engage in interactive behaviors, such as exploration and play, associated with positive experiences (15).

Potentially threatening situations can result in negative experiences such as anxiety and fear. These negative mental experiences likely result from a desire to avoid aversive situations (81, 82). However, when opportunities are provided for animals to build competence and exercise agency through choice and control, positive experiences (e.g., calm and relaxed) can replace these negative experiences (81, 82).

The precise valence and intensity of some individual mental experiences are still debated (e.g., boredom, helplessness) and likely vary depending on the individual’s life experiences and the length of time they are experiencing these feelings. Further exploration is needed to develop our conceptual understanding of these mental experiences. However, strategies to support agency and positive affective engagement focus on providing animals with opportunities to exercise a maximal ‘level of agency’.

## 6. Strategies to support agency and positive affective engagement

This section gives situational examples where animals can have competence-building agency and experience positive affective engagement. To illustrate this, we use two examples where opportunities for animals to exercise agency could be enhanced: sugar gliders kept as animal companions and greyhound dogs that race and are housed in kennels. Creating such opportunities for animals to exercise agency may require additional resources, such as space, equipment, or people’s time.

Assessing the welfare of animals using the Five Domains Model requires a systematic approach using all five domains. When experiences aligned to Domains 1 to 3 (e.g., hunger, pain) are sufficiently negative, animals may be less motivated to engage with opportunities for competence-building agency (84). In other words, without an overall experience of physical safety and health, an animal is less likely to engage in activities they might have found rewarding (83). However, given that this article focuses on the Behavioral Interactions (Domain 4), an abbreviated approach to identifying potential welfare impacts aligned with Domains 1 to 3 will be taken. This does not detract from the importance of a complete and systematic welfare assessment here; instead, it reflects a desire to focus specifically on elucidating the connections between Domain 4’s behavioral interactions with the environment, other animals and people, and positive welfare.

### 6.1. Sugar gliders housed in captivity as companion animals

Sugar gliders (*Petaurus breviceps* and *P. notatus*) are small, nocturnal, arboreal marsupials, native to parts of Australia and Oceania (38). In the wild, they live in colonies of 10–15 individuals in open forests and have an omnivorous diet of gum, sap, and insects (38, 85). This species spends most of the night active in tree branches and can glide up to 50 meters between trees (38, 85). They are highly active and maintain a territory of up to 1 ha in the wild (38). Although keeping these wild animals is restricted or prohibited in many places, Sugar gliders are an example of a non-domesticated animal commonly kept as companion ‘pocket pets’ in several countries globally, including the United States (85). They have an average lifespan of 7 years in the wild but can live up to 15 years in captivity (38). They have a paedomorphic appeal that likely triggers an instinctual human attraction – often described as the “baby schema effect” (86). A set of infantile (or neotenous) features, perceived as cute, evoke a nurturing response from humans, i.e., their small size (12 to 15 cm in length), facial features that are large in comparison to their round head, and large, dark, wideset eyes (38, 86). When kept as companions, they often present with veterinary problems associated with inappropriate housing, activity and diet, e.g., obesity (85).

#### 6.1.1. Domains 1 to 3

In captivity, welfare impacts aligned with Domains 1 to 3 are diverse. An inappropriate diet (Domain 1) is a common cause of sugar gliders presented to veterinary clinics (38). Many readily available diets show evidence of mineral and vitamin imbalances (38, 87). Diet-related conditions include malnutrition, obesity, osteodystrophy, and dental disease (38, 87). These will likely lead to mental experiences such as hunger, weakness, malaise, and pain. Sugar gliders tolerate temperatures between 18 and 32°C. Temperatures outside this range increase the risk of them experiencing various forms of discomfort and thermal extremes of chilling or overheating. Having sufficient space for spontaneous locomotion (Domain 2) and maintaining physical fitness (Domain 3) is also essential for positive welfare opportunities in Domain 4.

#### 6.1.2. Domain 4

Examples of positive behavioral interactions aligned with Domain 4 are further sub-categorized into interactions with the environment, other animals, and humans (Table 2).

**Table 2:** Examples of behavioral interactions (Domain 4) that can be provided to, and their utilization assessed in, sugar gliders housed in captivity (with aligned enrichment strategies from Table 1) that enable them to experience positive affective engagement (Domain 5) and their aligned agentic qualities.

Behavioral interactions	Agentic quality			
	Competence^1^	Choice^2^	Control^3^	Challenge^4^
Interactions with the environment				
A choice of materials^R^ that stimulate foraging behaviors^A^ (occupational, physical, nutritional)				
Aviaries of sufficient size^R^ to allow gliding^A^ (occupational, physical)				
A range of aviary items^R^ to encourage scurrying^A^, jumping^A^, climbing^A^, and gliding^A^ (occupational, physical)				
Able to avoid items^R^ in or near aviaries that may be perceived as a threat (sensory)				
Interactions with other animals				
Housed in groups of at least two individuals^M^ to enable social interactions^A^, and resting^A^ (occupational, social)				
Nests of sufficient size^R^ to allow individuals to huddle together^A^ (social)				
Space^R^ and housing design^M^ that allows them to avoid^A^ social interactions or predators that may be perceived as a threat (social)				
Interactions with humans				
Interactions limited to night-time only^M^ (occupational and social)				
Frequent quiet and calm handling with control over their engagement with the handling^M^ (social)				
Slow and controlled introductions to handlers^M^ to allow scent identification and familiarisation^A^ (social)				

Agentic qualities: ^1^Characteristics and skills developed through opportunities to exercise agency; ^2^Choice between two or more options; ^3^Able to decide when and how to interact; ^4^A variety of complex interactions that do not exceed an animal’s current competency level. Types of animal welfare indicators: ^R^Resource-based welfare indicators; ^M^Management-based welfare indicators; ^A^Animal-based welfare indicators.

##### 6.1.2.1. Interactions with the environment

In their natural habitat, sugar gliders are nocturnal and spend much of their awake time at night foraging for food, i.e., interacting with their environment. They use their long incisors to extract gum and strip bark from trees (38). When food is readily provided to captive sugar gliders, this not only increases their risk of developing obesity (Domain 1) but also reduces opportunities for them to perform feeding behaviors that build competence and would otherwise keep them occupied for extended periods (Domain 4) (38). Instead, materials that simulate foraging can be provided in captivity, e.g., holes drilled into non-toxic materials filled with food or other complex food toys (38). These are examples of occupational, physical, and nutritional enrichment strategies (Table 2) that allow sugar gliders to experience positive affective engagement.

Aviaries of sufficient size, particularly height, allow sugar gliders opportunities to glide between perches (38). These animals will also need branches- or rods arranged vertically and horizontally in their enclosure – to encourage scurrying, jumping, climbing, and gliding (38). Perches, swings, and ladders are valuable items in aviaries (38). Items resembling predators (e.g., clothing) should not be left where sugar gliders may perceive them as a threat, e.g., on top of cages, as this might limit their exploration and interaction with the full scope of available environment (38). For resting, a nest box should be provided in a suitably-sized aviary (85).

##### 6.1.2.2. Interactions with other animals

Sugar gliders are vulnerable on the ground and prefer to remain elevated (85). Sugar gliders are often kept individually in small bird cages with a suspended pouch as a nest (85). Sugar gliders in the wild are territorial and can become aggressive if not introduced carefully (85). The social nature of sugar gliders means that most guidelines recommend housing them in groups of at least two in captivity (38). Sugar gliders prefer to sleep huddled together, so nests should be large enough to allow co-habitation (38). Cats and other predatory species should not have access to sugar gliders (38). Although people may perceive sugar gliders as safe within an enclosure, probably, smelling the presence of predatory animals, such as cats, in the same space will impact their mental state and restrict behavior.

##### 6.1.2.3. Interactions with humans

Sugar gliders are nocturnal, so they should be handled at night when most active and not disturbed during daylight hours (38). Hand-reared sugar gliders handled quietly and calmly can develop into gentle companions (38). Scent has a vital role in social recognition in sugar gliders. For this reason, newly introduced and rehomed animals should be given time to recognize their handlers’ scents (38).

### 6.2. Racing greyhounds housed in kennels

Greyhound racing is a sport and gambling industry sector that relies on small groups of greyhounds running competitively out of starting boxes on a racetrack at speeds of around 70 kilometers per hour. The distinct life stages of greyhounds bred to race typically involve breeding, rearing, early education, training, racing, and leaving the industry. However, the industry’s practices have been subject to controversies and criticisms in the media and politics, with concerns about dog welfare and the business model’s ethics (88, 89). Globally, commercial greyhound racing is declining, remaining legal only in the United Kingdom, Ireland, Vietnam, Mexico, New Zealand and parts of the United States and Australia.

Practices across life stages tend to follow the same general model. Pups are born and stay with their mothers until weaned. By 12 weeks, they enter the rearing phase, which may occur in a paddock, kennel or barn environment. During this stage, they are often housed with some littermates. They enter early education schooling at approximately 1 year as the starting point for training and chasing. They enter residential kennels where they are housed individually and participate in training, trials, and sometimes sales or amateur racing before starting professional racing around 15 months of age. Dogs continue to live in residential kennels until they exit the racing industry, usually by 5 years old, if not before. They may leave racing due to injury or death on the racetrack, being retired, rehomed as a companion, or transitioning to a breeding role.

One of the main controversies surrounding greyhound racing, aside from the high rate of injuries and deaths on the track (88, 90), is the inadequate housing conditions and lack of compensatory environmental enrichment. Another issue raised is the inadequate socialization of puppies which impacts their ability to adapt as companions in new homes later in life, along with the apparent overbreeding and euthanasia or unknown fate of dogs considered surplus, known as *wastage* (91).

Overall, the controversies and criticisms surrounding greyhound racing have contributed to growing public awareness and scrutiny of the industry internationally. This has increased pressure on regulators, stakeholders, and industry insiders to address the welfare and ethical issues raised and consider alternative models for managing and caring for greyhounds in the sport.

#### 6.2.1. Domains 1 to 3

Greyhounds that race have increased nutritional demands (Domain 1). Nutrition should balance protein, fat, carbohydrate (including fiber), and vitamins. Protein is essential to support muscle use and growth. Extreme physical exertion likewise predisposes these dogs to dehydration (Domain 1). Inappropriate nutrition and hydration can lead to negative affective consequences such as thirst, hunger, weakness, and malaise of malnutrition. Appropriate hydration (Domain 1) is also necessary to control body temperature *via* panting (Domain 2). Systemic hyperthermia can result from exertion, hot environments, or an inability to cool effectively. Preventative health care is critical to optimize greyhound welfare (Domain 3). Disease prevention includes routine vaccination and parasite control. Training and racing intensity should match a dog’s current physical competence level. This means consideration should be given to maintaining training during downtimes or rehabilitative training following recovery from injury/illness. The critical importance of racetrack-related environmental features (e.g., kennel facilities and catch pen design) and appropriate pre-race warm-up activities to reduce the incidence of injury are reportedly overlooked during race meets (92, 93).

#### 6.2.2. Domain 4

Greyhounds that race spend a relatively brief period of their time budget running in one to two weekly races. Even if training, travel, handling, and kennelling are factored in, much of their time is spent outside engaging in racing-related activities. To counter the potential for boredom or frustration in the intervening time and to build competence (94), greyhounds should be provided with opportunities to exercise agency. Examples of opportunities for positive welfare aligned with Domain 4 are further sub-categorized into interactions with the environment, other animals, and humans (Table 3).

**Table 3:** Examples of behavioral interactions (Domain 4) that can be provided to, and their utilisaton assessed in, racing greyhounds housed in kennels (with aligned enrichment strategies from Table 1) that enable them to experience positive affective engagement (Domain 5) and their aligned agentic qualities.

Behavioral interactions	Agentic quality			
	Competence^1^	Choice^2^	Control^3^	Challenge^4^
Interactions with the environment				
Sufficient space^R^ to encourage free movement and play^A^				
Varied sensory inputs^R, e.g.,^ nosework (olfactory-based sniffing activities^AM^)				
Socialization and habituation^M^ to common household environmental stimuli^R^ to prepare for future rehoming as companion animals				
Interactions with other animals				
Access^M^ to congenial relationships with other dogs, e.g., the choice^A^ to live in pairs; regular play time^M^ in small groups with compatible individuals				
Able to avoid threatening situations^A, e.g.,^ sufficient space^R^ and responsive monitoring^M^ for threat avoidance				
Socialization^M^ and habituation to other animals				
Interactions with humans				
Reward-based training^M^				
Positive interactions^M^ with a variety of people^R^				

Agentic qualities: ^1^Characteristics and skills developed through opportunities to exercise agency; ^2^Choice between two or more options; ^3^Able to decide when and how to interact; ^4^A variety of complex interactions that do not exceed an animal’s current competency level. Types of animal welfare indicators: ^R^Resource-based welfare indicators; ^M^Management-based welfare indicators; ^A^Animal-based welfare indicators.

##### 6.2.2.1. Interactions with the environment

Designated spaces provided beyond the primary housing or kennel facility can allow greyhounds to explore and interact with their surroundings. Outdoor areas featuring a diversity of elements and substrates (e.g., grass, sand, trees, gravel, etc.) facilitate physical activities that promote fitness and allow for the expression of social (e.g., turning and jumping while engaged in social play) and other behaviors (e.g., digging) (95). Indoor spaces can be provided to preview the home environment (e.g., appliances and furniture) that retired dogs should transition to, allowing dogs to navigate and adapt to different challenges and settings that will set them up to succeed as competent animal companions beyond their time in racing (96).

Within their primary housing and transportation containment, sufficient space for easy stretching, lying down in full extension, and turning around should be ensured. This will enable greyhounds control to move comfortably. Providing multiple resting areas (e.g., elevated resting platforms and beds at ground level) allows dogs to choose how they utilize the space available to them (97). These provisions enable them to adjust their body positions, express their preferences, and exercise agency. Greyhounds may reposition bedding material to their liking, another way to exercise control. Providing more space to greyhounds promotes movement, reducing the likelihood they will experience affects such as frustration or discomfort. However, increased space alone is unlikely to offer sufficient agentic opportunities for positive welfare (98).

Interactive sensory stations can be provided in both indoor and outdoor spaces. These feature various scents, textures and objects for greyhounds to investigate and safely interact with. Based on their individual preferences and curiosity, such stations offer the dogs a choice as to what they engage with. Additional opportunities for positive experiences can come from devices such as puzzle toys and treat-dispensing toys, which engage greyhounds in challenge, both physically and cognitively (99). The complexity of spaces, objects, sensory stations and other novel objects should be gradually increased to support the animals’ agentic choice and control to support the development of competence.

##### 6.2.2.2. Interactions with other animals

Facilitating supervised interactions with other dogs allows greyhounds to develop and engage in appropriate social behaviors and establish positive social connections. Social connections provide opportunities for positive experiences through companionship, social bonding, and play (100–102). These experiences can also provide the greyhounds with exercise and a sense of comfort and security, promoting relaxation. Social housing, where compatible dogs live in pairs or small groups, facilitates social interactions. One way this can be achieved in a kennel facility is by enabling access between adjoining kennel runs so that multiple dogs can choose to be together or separate. Adequate space to comfortably accommodate the pair or group of dogs must be available in any kennel run if this strategy for shared housing is adopted.

Historically, greyhounds that race have been identified as having relatively poor socialization practices (103, 104). This can be related to isolated rearing occurring in rural locations and limited resourcing for active practices to adequately compensate. Social interactions with various other dogs help puppies learn and develop appropriate social and communication skills with conspecifics (105). Play groups that allow greyhounds to interact with other dogs of various breeds, sizes, ages and temperaments will expand their social skills’ flexibility (i.e., competence) in response to dogs they meet throughout their life.

Positive experiences with other animals, both large and small, allow dogs to learn how to interact appropriately with different animals (106). This further develops their social skills and competence in multi-species environments, which is particularly relevant for successful rehoming following racing. Opportunities to interact with other animals can be provided with appropriate supervision and choice. In this way, individual dogs can exercise their agency, approaching and engaging with other animals (e.g., meeting a horse through a fence while on lead). Allowing greyhounds to learn to relate socially with other animals in a supportive manner is a challenge that can contribute to their overall competence. Foster programs in private homes (i.e., as often undertaken in working dog programs such as detection or guide dog rearing) during puppyhood and throughout the time a greyhound is racing may provide essential respite from the kennel environment (107) and alternative experiences to interact with a variety of animals and people (108, 109).

##### 6.2.2.3. Interactions with humans

Ensuring that interactions with people, such as grooming and play sessions, are positive for greyhounds builds trust and promotes healthy attachment between the dogs and their caregivers (110, 111). For example, interactive play sessions between people and greyhounds can be undertaken using toys, agility equipment, or flirt poles. Such sessions enable the dogs to exercise choice in initiating and controlling their level of engagement while also challenging them physically and cognitively, promoting competence. Positive reinforcement training should form the basis of all foundational interactions between humans and greyhounds (112, 113).

Training activities can offer both cognitive and physical challenges relating to learning new behaviors, problem-solving, and overcoming obstacles of increasing complexity. With experience, this builds canine confidence in interacting with people, and their competence can increase. Dogs learn through every interaction that their behaviors directly influence the outcomes they receive, providing the individual animal with control in their training exercises. Greyhounds should be granted the choice to actively opt-out of training sessions if they do not wish to engage in the behaviors or with the equipment that will earn them rewards, providing them with control over their actions. Providing greyhounds with individual attention from people also allows for personalized interaction and the development of positive social bonds. This also facilitates the personalisation of training and care practices in a manner that can safeguard against fear, anxiety, or frustration.

It is important that greyhounds who race are able to meet a variety of people during puppyhood and their time in racing (114, 115). This include people of different ages, heights, appearances, and sex. Facilitating good socialization and ongoing experiences with a diversity of people allows greyhounds to interact positively (competently) with humans during and after their time in racing, a desirable trait for dogs.

### 6.3. Supporting agency and positive affective engagement

The two scenarios presented above are not intended to be exhaustive representations of how opportunities for agency could be supported in each. Instead, they have been used to illustrate how animals can be given opportunities to exercise agency in various contexts. Choice, control, and challenge represent agentic qualities that appropriate human care can provide, while competence likely results from these opportunities. Conversely, umwelt and affordances are agentic qualities not directly impacted by human care – so they have not been included in Tables 2, 3. They represent an animal’s unique perceptual and effector world (umwelt) and their perception of what their environment offers them (affordances). Umwelt, affordances, and competence represent agentic qualities that need further exploration to identify potentially relevant positive welfare indicators.

While our evaluation of negative impacts in Domains 1 to 3 for each case study scenario focused on the potential mental experiences that might be inferred from conditions in each domain (e.g., hunger, weakness, and pain), this was not the case for Domain 4 (Behavioral Interactions) and positive welfare. Instead, we found it more beneficial to evaluate opportunities for agency to be exercised by considering agentic qualities of choice, control, and challenge that could be provided to the animal(s). In essence, we evaluated features of positive affective engagement (i.e., the collective term) rather than specific named positive mental experiences. This approach provides a means of systematically evaluating options to provide animals with opportunities to exercise agency. It may also help risk-averse animal welfare scientists cross the positivist-affective divide.

One flaw with our approach to evaluating positive welfare is that many behavioral interactions in our two scenarios mapped across similar or identical agentic qualities (Tables 2, 3). Therefore, detailed comparisons between interactions might be challenging to perform. An alternative approach might involve some indication of how strongly each agentic quality is exercised by a behavioral interaction being offered or occurring for the animal(s). For example, a behavioral interaction might offer an animal the ability to exercise a high level of choice, low control, and moderate challenge (Table 4). This behavioral interaction could then be compared against the agentic qualities of another interaction and this comparison might allow us to account for the interests of an individual animal or species. A non-numerical score could also be assigned to indicate how confident the rater is in assigning the strengths of these agentic qualities to the behavioral interaction (Table 4), i.e., to indicate the strength of the evidence used to assign the agentic score (116, 117).

**Table 4:** Opportunities for positive interactions (Domain 4) can be provided to an animal, and their utilization assessed so that the animal’s experience of positive affective engagement (Domain 5) can be inferred.

Behavioral interactions	Agentic quality		
	Choice	Control	Challenge
Example behavioral interaction	**	***	*

The agentic qualities have been color-coded for each behavioral interaction being assessed. These colors represent how strongly each quality is exercised by the behavioral interaction being offered or occurring (e.g., green = high; yellow = moderate; red = low). Asterisk(s) could be used to indicate the degree of confidence a rater has in assigning the color code for each agentic quality – from low (*) to high (***).

Competence has not been included in Table 4 as this was the agentic quality that mapped across most behavioral interactions in our scenarios. The agentic qualities of choice, control, and challenge represent opportunities for agency that can be provided by human care and management decisions, while competence is the potential result of these opportunities. Therefore, including competence did not provide additional information beyond that provided by the other three agentic qualities. However, future iterations could see competence included with sub-categories of physical and cognitive/mental competence to distinguish the types of competence that might result from each behavioral interaction (26, 34, 65).

In the two scenarios presented above, we have focused on opportunities for positive behavioral interactions. There is also scope to assess how well animals utilize these opportunities (15). An animal can be given opportunities to exercise agency (i.e., human care and management). Still, the animal’s actual utilization of these opportunities determines whether or not they experience positive affective engagement (i.e., positive animal welfare). The approach in Table 4 might be used as a staged evaluation, where Stage 1 involves identifying opportunities for behavioral interactions, and Stage 2 is where the animal’s utilization is assessed (15). However, animal utilization might be challenging to assess given that a lack of ‘utilization’ does not imply agency is not being exercised, i.e., an animal not interacting with an opportunity provided to them is still exercising agency through choice and control (57, 65). This area of evaluation and continuous improvement in offering greater agentic opportunities to animals under human care and management is an important consideration for future focus.

Future consideration should also be given to best practice communication with stakeholders (e.g., animal caretakers, industry bodies, regulators, policymakers, and the general public) about agency and positive animal welfare (118, 119). Translating theoretical and research findings to meaningful change for animals under human care often depends upon effective communication and subsequent human behavior change.

## 7. Conclusion

Animal welfare is a complex and multi-disciplinary field that encompasses the subjective mental experiences of animals. Focusing on mental experiences is becoming increasingly important in contemporary animal welfare science, as it aligns with other aspects of safeguarding and animal welfare assurance, such as ethics, policy, and laws. However, assessing animal welfare based on mental experiences can pose challenges, as they are subjective and cannot be directly measured. The concept of agency represents a new frontier in animal welfare assurance, as it allows us to consider how animals can be given opportunities to experience positive welfare by engaging in voluntary, self-generated, and goal-directed behavior that they are motivated to perform. This article argues that agency is a concept that straddles the positivist-affective divide and represents a way forward for discussions about and opportunities for positive animal welfare. Understanding the relationship between an animal’s welfare and their ability to exercise agency can be illustrated through Domain 4 (Behavioral Interactions) of the Five Domains Model. Overall, the concept of agency provides a promising approach to understanding and improving the welfare of animals.

## Author contributions

KL: Conceptualization, Writing – original draft, Writing – review & editing. MH: Conceptualization, Writing – review & editing. MC: Conceptualization, Writing – review & editing.
